# Quantitative assessment of asbestos fibers in some normal and pathological extra-abdominal tissues—a scoping review

**DOI:** 10.1186/s12995-023-00392-4

**Published:** 2023-11-09

**Authors:** Francesca Graziosi, Paola Caffaro, Mattia Bonetti, Francesco Roccuzzo, Samantha Rota, Paolo Boffetta, Yohama Auxiliadora Caraballo-Arias, Carlotta Zunarelli, Nataliia Danilevskaia, Francesco Saverio Violante

**Affiliations:** 1 Department of Medical and Surgical Sciences, Alma Mater Studiorum University of Bologna, 40138 Bologna, Italy; 2 School of Occupational Medicine, Alma Mater Studiorum University of Bologna, 40138 Bologna, Italy; 3 Stony Brook Cancer Center, Stony Brook University, New York, NY 11794 USA; 4 Department of Family, Population and Preventive Medicine, Renaissance School of Medicine, Stony Brook, NY USA; 5 IRCCS Azienda Ospedaliero-Universitaria Di Bologna, 40138 Bologna, Italy

**Keywords:** Asbestos fibers, Amphiboles, Chrysotile, Electron microscopy, Lymph node, Scoping review

## Abstract

**Abstract:**

**Background:**

Asbestos is a mineral present in nature and it has been used for years in numerous settings. Asbestos enters the bloodstream and lymphatic system mainly through breathing.

**Objectives:**

Studies with asbestos fiber’s quantification in human tissues are scarce except for the lung. This article summarizes asbestos studies in some extra-abdominal tissues.

**Methods:**

A scoping review of articles that quantified asbestos fibers in extra-abdominal tissues (lymph nodes, pharynx, larynx, trachea, heart) by electron microscopy (Scanning—SEM or Transmission—TEM) was performed.

**Results:**

The 10 studies selected comprised 52 cases, out of whom 108 samples were analyzed. Mostly samples were lymph node tissues (102), followed by larynx (3) and myocardium (3). No studies were found that determined the presence of asbestos in the pharynx or trachea. The concentration of asbestos fibers detected in the lymph nodes was from 0.003 million fibers per gram of dry tissue (mfgdt) up to 7400 mfgdt, in the larynx the range was from 0.5 mfgdt up to 3.6 mfgdt, in myocardium no asbestos fibers were detected.

**Discussion:**

The studies included were heterogeneous in terms of case and sample characteristics and analytical techniques. As subjects exposed to asbestos are often positive for fibers in thoracic lymph nodes, we suggest that whenever a human tissue sample is analyzed for asbestos presence, the relevant draining lymph node should be concomitantly studied.

## Introduction

Asbestos is a mineral present in nature and for years it has been used in various fields for its insulation properties. There are two categories of asbestos fibers based on their shape and mineralogic characteristics. Serpentine fibers, of which chrysotile is the main commercial variety, are long, curly strands, whereas amphibole fibers (crocidolite, amosite, tremolite, and others) are long, straight, needle-like structures [33].

Since various types of asbestos are present in almost the entire earth's crust [2], which was before industrial use of asbestos started, environmental exposure to geological resources [5, 25] is an important source of natural exposure.

When the use of asbestos was widespread, exposure occurred in a variety of occupational and nonoccupational settings, including mining and milling of the fibers, and use of this material in a variety of manufacturing industries such as asbestos-cement materials, friction materials, insulation, shipbuilding, textile [1, 14, 26, 36]. Non-occupational exposure to airborne asbestos occurred mainly via residence near industrial sources, contact with soiled clothes brought home by asbestos workers [20] and fibers spread from renovation or demolition of asbestos containing buildings [11, 31].

Since asbestos has been recognized as a potent carcinogen [6, 22], the use of asbestos has been restricted in most parts of the world, and many high income countries are attempting to reduce the presence of the mineral through removal interventions [37].

Currently, it is estimated that many millions of people are still environmentally exposed to asbestos worldwide, even in countries that banned its use [15].

The presence of asbestos in human tissues has been documented since many years in the scientific literature through light and electron microscopy. Some scientific articles reported asbestos fibers counted using light microscopy [24, 29], others using electron microscopy. The greater resolving power and the ability to obtain information on the chemical composition and crystal structure of the fibers of electron microscopy compared to light microscopy have made this technique the most accurate for quantifying asbestos fibers in human tissues [12].

The penetration of asbestos into the human body occurs mainly by inhalation [22, 23] and from the airways asbestos reaches the bloodstream and the lymphatic system [8, 18, 19, 32]. Not overlooking asbestos is present in drinkable water [39] and possibly in food, another way of entry is through digestive system. For all these reasons, it is certainly important to have a clear picture of the presence of asbestos fibers in the body.

As a part of this endeavor, we have already published two reviews on asbestos fibers in pleural and peritoneal tissues respectively [3, 4], and prepared two other reviews on asbestos fibers in abdominal organs and female reproductive organs: thus we undertook this review to address the presence of asbestos in extra-abdominal tissues, whereas the presence of asbestos in the lung certainly deserves a systematic review and meta-analysis due to the large number of studies available.

## Methods

As this was conceived as an exploratory endeavor, PRISMA extension for scoping reviews was followed for summarizing the literature [35]).

The search was conducted on different databases, such as Pubmed, Scopus, Cochrane and Web of Science. The string used for the selection of the articles was: “(Asbestos AND electron*) AND ((pharyn*) OR (laryn*) OR (trachea*) OR (lymph*) OR (heart* OR myocard*))” on 26 April 2023.

The inclusion criteria for this review were:

- articles written in any language regardless of the publication date and of the type of the study;
- articles reporting a quantification of asbestos fibers in human tissues (pharynx, larynx, trachea, heart, myocardium and thoracic lymph nodes) with electronic microscopy, in subjects with occupational and/or non-occupational exposure.

The exclusion criteria were:

- articles not reporting a quantitative measure of the number of asbestos fibers found or reporting a measure by techniques other than electron microscopy;
- studies in animals or in tissues other than those listed on inclusion criteria.

**Fig. 1:**
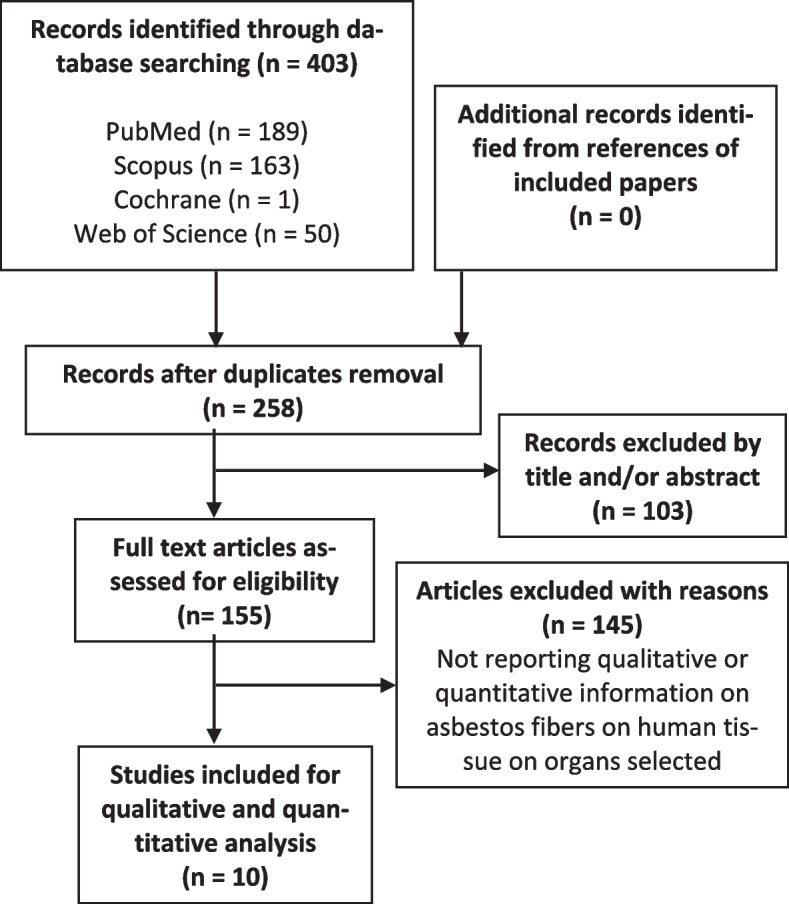
Flowchart showing the selection process of the articles included in the scoping-review

As reported in Fig. 1, the search yielded 258 articles, which have been independently evaluated by the authors. The references of each relevant article were searched, yielding no more papers fitting our inclusion criteria. Finally, we identified 10 papers that fitted our inclusion criteria for the scoping review. The 10 studies included were case series published between December 1979 and September 2014. We could not locate any article fitting our inclusion criteria published in the last 9 years (from 2014 to 2023).

## Results

The 10 studies included determined the number of asbestos fibers in specific tissues (larynx, myocardium and thoracic lymph nodes) on 52 subjects, from whom 108 samples of tissue were extracted. No article met the inclusion criteria of the scoping review for pharynx and trachea.

The samples were mostly obtained from autopsies and only one study analyzed samples from biopsy. Few data for each type of tissue were obtained from the analysis of all included articles, not allowing a comparison of asbestos fibers found in specific tissues in relation to different population groups (i.e., cases with asbestos-related diagnoses compared to general population).

A description of the subjects and conditions included in the studies was reported in Table 1.

**Table 1:** Description of the subjects and conditions in the included studies

Reference	Number of cases	Type of diagnosis
[21]	2 biopsies	1 vocal polyp 1 laryngeal carcinoma
[29]	3 autopsies	1 asbestosis 1 pleural mesothelioma 1 lung cancer
[7]	8 autopsies	4 asbestosis 1 laryngeal cancer 3 other causes
[34]	1 autopsy	1 lung cancer
[28]	3 autopsies	2 asbestosis 1 other cause
[8]	21 autopsies	Ongoing autopsies for several cases of death
[9]	1 autopsy	1 pleural mesothelioma
[10]	11 autopsies	8 mesotheliomas 2 lung cancers 1 paranasal sinus cancer
[11]	1 autopsy	1 malignant mesothelioma
[16]	1 autopsy	1 pleural mesothelioma

As shown in Table 2, in most of the studies included in this review the analysis and quantification of asbestos fibers was carried out using TEM with EDX. This technology may detect both short (< 5 µm) and longer/thin fibers [9]. However, the counting schemes chosen by the authors are different. For example, Gordon et al. [16] counted only long fibers (> 5 µm) although, as stated by the authors themselves, they observed even the smallest ones using TEM.

**Table 2:** Detection limit (DL) of asbestos fibers, expressed in millions of fibers per gram of dry tissue (mfgdt) and technologies used

Reference	Detection limit for amphiboles (mfgdt)^a^	Detection limit for chrysotile (mfgdt) ^a^	Technology used	Dimension limit for fiber counting (length in µm)
[21]	0.5	0.5	TEM with EDS	Not reported
[29]	0.1	0.1	SEM	≥ 5
[7]	0.003	0.003	TEM with EDX	≥ 0.5
[34]	0.1	0.1	SEM	> 1
[28]	0	0	TEM with EDS	Not reported
[8]	0.11	0.11	ATEM with EDX and SAED	≥ 0.5
[9]	0.65	0.65	ATEM with EDX	< 5
[10]	0	0	ATEM with EDX and SAED	≥ 0.5
[11]	0.5	0.5	ATEM with SAED	> 3
[16]	0.021	0.021	TEM with EDS and SAED	> 5

*TEM* Transmission electron microscope

*EDS* Energy dispersive X-ray spectroscopy

*SEM* Scanning electron microscope

*EDX* Energy dispersive X-ray analysis

*ATEM* Analytical transmission electron microscope

*SAED* Selected area electron diffraction

^a^When the DL was not specified the lowest value observed is reported

Thus, the comparison of asbestos fibers concentrations obtained from different studies over time, is difficult and limits information on the number, type and size of fibers detected.

### Lymph nodes

As reported in Table 3, a quantitative assessment of asbestos fibers of the lymph nodes was analyzed in 9 studies from 1979 to 2014 [7–11, 16, 21, 29] which included 48 subjects. The cases were mostly men (40 subjects) from 15 to 78 years old, while women represented a smaller sample (8 subjects) with ages from 12 to 82 years old.

**Table 3:** Asbestos fibers found in lymph nodes (for additional details the readers are referred to the synthesis of the studies in the text)

Reference	Number of subjects / number of samples	Type of tissue analyzed	Type of asbestos exposure	Type of asbestos found (number of samples with/without fibers)	Range of asbestos fibers mfgdt^a^
[21]	1/1	Mediastinal lymph node	Occupational	Total (1/0)	6.4
[29]	3/3	Hilar lymph node	Occupational	Total (3/0)	0.1- 9.01
[7]	8/8	Tracheal lymph node	Occupational	Amphiboles (8/0)	0.003—1.7
				Chrysotile (6/2)	Not detected—0.018
[34]	1/1	Hilar lymph node	Occupational	Crocidolite (1/0)	7400
[8]	21/21	Peritracheal lymph node	Unexposed	Amphiboles (13/8)	0.11—1.7
[9]	1/1	Hilar lymph node	Environmental (smoker of cigarettes with asbestos in the filters)	Amphiboles (1/0)	14.32
[10]	11/65	Thoracic lymph node	Occupational	Chrysotile (3/62)	0—1.8
				Amphiboles (59/6)	0—262.25
[11]	1/1	Peribronchial lymph node	Occupational	Libby amphiboles (1/0)^b^	0.5
[16]	1/1	Thoracic lymph node	Unexposed	Amphiboles (1/0)	0.127

^a^When original data were reported for wet tissue, the results were multiplied by 10 to convert them to dry tissue

^b^The Libby amphiboles definition is reported in the text

The total of the samples analyzed was 102, of which 88 had asbestos fibers (mostly amphibole types). At least one type of asbestos fibers (amphiboles or chrysotile) was found in each of the samples taken from cases with occupational exposure, as might be expected. However, the data show the presence of asbestos fibers even in most of those not occupationally exposed, highlighting the theme of environmental exposure or the use of products contaminated or containing asbestos fibers [8, 9, 16].

From the range of samples with asbestos fibers, it is not possible to deduce a uniform pattern of distribution, with extreme values that are 0.003 mfgdt and 7400 mfgdt (a particularly high value found in an asbestos sprayer).

Most studies observed fibers shorter than 5 µm (average lengths observed in different studies were in the range from 1.27 µm to 4.12 µm).

Hirsch et al. [21] analyzed one sample of mediastinal lymph node from a case of a 51-year-old man (smoker) who had an occupational history in a welding factory. In the same case, he previously took a biopsy sample of a vocal polyp in which asbestos fibers were found (see below). It was found by TEM with energy dispersive X-ray spectroscopy (EDS) that the amphibole type was predominant (concentration of 6.4 mfgdt). The detection limit was 0.5 mfgdt for asbestos fibers. The mean length of the fibers detected was 3.3 µm, with a mean diameter of 0.15 µm.

Roggli and Benning [29] studied histologic sections of hilar lymph nodes from 3 autopsies. The samples were taken from men who had an occupational history of exposure to asbestos and who had been diagnosed with asbestosis. The observation of asbestos fibers was performed by SEM, with a detection limit of 0.1 mgfdt. The range was from 0.1 mfgdt to 9.01 mfgdt and included only fibers with a length of 5 µm or greater. No other information about dimension or diameter was reported.

Dodson et al. [7] performed quantification of asbestos fibers in tracheal lymph nodes samples from 8 subjects who were occupationally exposed. Medical histories of the shipyard workers showed several diseases such asbestosis, lung cancer, lung fibrosis, emphysema and cardiovascular pathologies. Subjects were 58 to 82 years of age at the time of autopsy. The analysis was conducted by TEM with energy dispersive X-ray analysis (EDX) and the detection limit was 0.003 mfgdt. Asbestos fibers were defined as fibers with length >  = 0.5 µm and sides roughly parallel to the fiber axis, conforming to a 3:1 aspect ratio. In most of the subjects the numbers of asbestos fibers per gram of dry tissue from the tracheal lymph nodes exceeded the numbers per gram in the lung (belonging to the same cases). Amphibole fibers were detected in all lymph node samples (with a concentration ranging from 0.003 mfgdt to 1.7 mfgdt). The average length of amphibole fibers was 2.22 ± 3.36 µm and the average width was 0.21 ± 0.12 µm. Chrysotile fibers were identified in 6/8 samples (concentration ranging from not detected to 0.018 mfgdt) and 2/8 did not present chrysotile fibers. The average length of chrysotile fibers was 1.27 ± 1.07 µm and the average width was 0.08 ± 0.06 µm.

Tossavainen et al. [34] analyzed tissue of hilar lymph node from an autopsy of a worker who had died from lung cancer and was employed as crocidolite sprayer. Other samples of tissues were taken from the same case. The sample was observed by SEM with a detection limit of 0.1 mfgdt. Lymph node tissue had the highest asbestos fiber level (7400 mfgdt), followed by lung parenchyma, visceral pleura and parietal pleura. Asbestos fibers > 1 µm in length and > 0.3 µm in diameter were counted. The median dimensions of crocidolite fibers were 2.0 to 3.0 µm for length and 0.12 to 0.13 µm for diameter with no obvious difference between various tissues. Size of crocidolite fibers in hilar lymph nodes had a length measure of 1 to 6.0 µm with a median of 2.2 µm and a diameter measure of 0.05 to 0.30 µm with a median of 0.13 µm. For comparison, asbestos fibers were also counted from lung parenchyma, lymph nodes, and kidney cortex in ten autopsied control cases collected from an unselected series of sudden Finnish male deaths. The concentration in the lymph nodes of the controls was in the range from 5 to 81 mfgdt with a mean value of 21 mfgdt. The data of these control cases were not reported in the tables since information was not available in the article.

In 2000, Dodson et al. [8] determined the concentrations of asbestos fibers in the lymph nodes of the general population. Samples of peritracheal lymph nodes and lung tissue from 21 individuals non-occupationally exposed to asbestos were taken at autopsy and were analyzed by analytical transmission electron microscopy (ATEM) and EDX. The detection limit was 0.11 mgfdt. Amphiboles fibers were detected in 13/8 samples with a range of 0.11 mgfdt to 1.7 mgfdt. The most common type of asbestos found in lymph nodes was anthophyllite, followed by tremolite and amosite. No chrysotile fibers were detected in the tissue analyzed. All fibers with length 0.5 µm or more and a 3:1 aspect ratio were detected. The mean length for asbestos fibers was 2.52 µm and the mean width was 0.27 µm.

Dodson et Hammar [9] described a case of asbestos burden in hilar lymph node tissue in a 67-yr-old woman who died from mesothelioma and whose only known exposure to asbestos was from smoking crocidolite asbestos-containing filtered cigarettes. From 5 original samples, after the preparation procedures applied on the tissue, the counting of asbestos fibers was made on a unique sample of 0.0094 g of dry tissue. The lymph nodes contained 14.32 mgfdt. The detection limit for asbestos fibers was 0.65 mgfdt and only amphibole fibers were detected (crocidolite, tremolite, anthophyllite). Asbestos fibers < 5 µm in length or longer were counted. The length of the asbestos fibers was from less than 4 µm to 12 µm.

Dodson et al. selected tissue samples from 11 males with a history of asbestos exposure [10]. Their cause of death was mostly from lung cancer and mesothelioma and, in one case, a left paranasal sinus squamous cell carcinoma. The study evaluated concentration of asbestos fibers in lymph nodes taken from locations anatomically defined according to the Naruke lymph node map [27]. All fibers equal to or greater than 0.5 µm in length were analyzed with ATEM equipped with EDX and selected area electron diffraction (SAED). Amphiboles were detected in 59/6 samples, ranging from 0 to 262.25 mgfdt. Asbestos fibers burden of the various lymph nodes from each case showed a preponderance of amphiboles, particularly amosite and crocidolite. The second most commonly encountered asbestos type was tremolite and anthophyllite. Chrysotile fibers were detected in 3/62 samples with a range of 0 to 1.8 mgfdt. The authors reported as a geometric means of total asbestos fibers a value of 3.52 µm in length and a value of 0.15 µm in width.

Dodson et al. [11] presented a case study reported on tissue burden of fibrous dust in an 81-year-old woman with diffuse malignant mesothelioma. Her home was built and renovated with Libby vermiculite. Libby vermiculite is a material with possible contamination of asbestos. The latency period from time of exposure was over 50 years. Three pieces of peribronchial lymph node were taken from autopsy, but only one sample was subjected to laboratory procedure for specific analysis in ATEM. The detection limit matched the lower value reported of 0.5 mfgdt. The data from the high magnification scan of the lymph node preparation were found to contain 1 tremolite asbestos fiber, 1 anthophyllite asbestos fiber, and 6 Libby amphiboles. The 6 Libby amphiboles were equivalent to 0.5 mfgdt. The identification of the only fibers with length > 3 µm was conducted in the area scanned. Libby amphiboles found in all the samples analyzed (lung and lymph node) showed a length from 5 to 68 µm with an average length of 20.7 µm.

Gordon et al. [16] analyzed lymph nodes from a woman with pleural mesothelioma and with no other known exposure to asbestos other than her use of a cosmetic talcum powder. The brand of talcum powder in question contained asbestos, and the application of its talcum powder released inhalable asbestos fibers. It should be remembered that the talcum powder itself it is not always contaminated, however it may contain asbestos impurities [30]. The location of the lymph nodes was not specified, but it can be deduced a thoracic allocation as it is reported in the study that lung and lymph node tissue were removed at autopsy. TEM analysis of the tissue revealed amphibole asbestos fibers with a value of 0.127 mfgdt, with a limit of detection of 0.021 mfgdt. The amphiboles were identified by EDS and SAED analysis as anthophyllite and tremolite and they were seen in a ratio of 5:1 anthophyllite: tremolite. Many fibers less than 5 µm in length were not counted, only fibers 5 µm or more in length and with aspect ratios of 10:1 or greater.

### Larynx

A quantitative assessment of asbestos fibers of the larynx was analyzed in 2 studies [21, 28] as reported in Table 4.

**Table 4:** Asbestos fibers found in larynx (for additional details the readers are referred to the synthesis of the studies in the text)

Reference	Number of subjects / number of samples	Type of tissue analyzed	Type of asbestos exposure	Type of asbestos found (number of samples with/without fibers)	Range of asbestos fibers mfgdt^*^
[21]	2/2	Larynx	Occupational	Total (2/0)	0.5—3.6
[28]	1/1	Larynx	Occupational	Total (0/1)	0

^*^When original data were reported for wet tissue, the results were multiplied by 10 to convert them to dry tissue

In the study of Hirsch et al. [21] one sample was taken from a biopsy of a case with vocal polyp and one sample from a case with laryngeal carcinoma. Asbestos fibers were detected in the 2 samples deriving from the 2 patients, no case was found without asbestos fibers. The range of asbestos fibers was from 0.5 mfgdt to 3.6 mfgdt. Both patients were occupationally exposed to asbestos. Both chrysotile and amphibole fibers were found. In one case, mostly chrysotile type fibers were found, in the other case mostly amphiboles. The mean length of the fibers detected ranged from 2.4 µm to 3.4 µm, with a mean diameter from 0.08 µm to 0.14 µm.

In 1997, Pollice et al. [28] reported the concentration of asbestos fibers in 7 samples of extra-abdominal tissues from 3 cases. Two cases had pulmonary asbestosis with a history of asbestos exposure and one control case died for other cause without asbestos exposure. Autoptic samples of the three men were analysed by TEM and energy dispersion spectrometry (EDAX). The analytical procedures described in the study do not specify the detection limit, but the minimum concentration detected was 0.1 mfgdt. In both cases and in the control case, the fibers in larynx tissues were absent or not detectable.

### Myocardium

Myocardium has been only analyzed in the article by [28], as shown in Table 5. Three samples were taken from 3 autopsies, one for each case. No asbestos fibers were found in the histological sections of the three reported cases [28], although fibers were counted in the lungs and pleura of the occupationally exposed cases.

**Table 5:** Asbestos fibers found in myocardium

Reference	Number of subjects / number of samples	Type of tissue analyzed	Type of asbestos exposure	Type of asbestos found (number of samples with/without fibers)	Range of asbestos fibers mfgdt^*^
[28]	2/2	Myocardium	Occupational	Total (0/2)	0
	1/1	Myocardium	Unexposed	Total (0/1)	0

^*^When original data were reported for wet tissue, the results were multiplied by 10 to convert them to dry tissue

## Discussion

Most of the included articles in this review had as a primary objective the determination of asbestos fibers within the lung tissue. Besides the analysis of the lung, other tissues were analyzed by the authors, with or without finding asbestos fibers. There are few studies focusing directly on the tissues selected for this review [8, 21, 29].

The extra-abdominal tissues most analyzed were lymph node samples. Asbestos fibers were detected in lymph nodes in 88/102 available samples. The localization of the glands was always in the thorax and, when the type of asbestos fibers was reported, this was mostly amphiboles.

In a few studies [21, 29] the fibers found in lymph nodes were not different regarding order of magnitude and type from those found in pulmonary parenchyma.

Data from the study of Dodson et al. [7, 8] show that the number of asbestos fibers per gram of dry tissue in the lymph nodes exceeded the number per gram in the lung. According to the authors, this suggests that the accumulation of fibers over time can result in an appreciable burden in extrapulmonary sites. Similarly, Tossavainen et al. [34] reported a higher concentration of crocidolite fibers in the lymph node than in the lung parenchyma, even if the size and aspect ratio of the fibers in the extrapulmonary tissues were the same as those found in the lung, indicating that the translocation processes were rather unselective regarding fiber dimensions.

Some authors who have found more fibers in the lymph nodes than in the lungs suggested that stations in the lymphatic system represent a "reservoir of retained material", particularly in those subjects exposed for a longer period [8, 10]. Others claimed that while lung tissue is subjected to clearance mechanisms that may be highly efficient, clearance from lymphatic tissue is relatively slow [13].

Two larynx samples were positive for asbestos fibers with both amphiboles and chrysotile types found [21]. The authors suggest fibers can penetrate laryngeal tissue either from the external surface of the larynx or from the blood or lymphatic circulation. The data showed that intra-laryngeal fibers are not different regarding type and size from those found in pulmonary parenchyma [21].

Qualitative assessment also established the presence of asbestos fibers in laryngeal tissue [24, 38] but the articles were not considered in this scoping review as they reported only a qualitative quantification of asbestos fibers.

No positive samples were found for myocardium and heart.

No articles met the inclusion criteria of the scoping review for trachea and pharynx.

Finally, it is necessary to highlight some limitations of this review. This scoping review identified only 10 studies, which are very heterogeneous in terms of case and sample characteristics, and did not allow a formal comparison of the results across them. Each study focused on diverse groups of people according to age, gender or occupation, exhibiting varying levels of asbestos-related illnesses. The cases examined also differed in terms of occupational or environmental asbestos exposure.

Heterogeneity also arises from the selection of the analytical instrument. Electron microscopes (SEM and TEM) have different resolving power. The TEM detects more fibers than the SEM, which however reveals more fibers than an optical microscope [17]. This leads to a difference in the counting of asbestos fibers, furthermore the author's choice to use different cut-offs for the detection of asbestos fibers must be considered. In fact, there is substantial variation among studies identified in fiber counting procedures (see Table 2). For example, some studies only count fibers longer than 5 µm [16, 29], one study counts fibers longer than 1 µm [34], whereas others count all observed fibers [7–10]. Variation in counting techniques may limit the comparison of results from different studies, thus limiting information for asbestos-associated disease research and exposure assessment. Therefore, we recommend that standardization of counting techniques be developed by considering fiber cut-offs (size parameters, including shorter and thinner fibers) each time an analysis is performed.

In conclusion, the search for fibers in the selected extra-abdominal organs resulted in the detection of asbestos mainly in the lymph nodes of occupationally exposed subjects. However, fibers were also detected in the lymph nodes of unexposed subjects even where other organs (lungs) did not present asbestos fibers [8].

As few data were available, we may not suggest new pathway hypotheses, but we underline the role of the lymphatic system in the transport, distribution, and accumulation of fibers, especially of small size fibers.

Therefore, we suggest that whenever a human tissue sample is analyzed for asbestos presence, the relevant draining lymph node should also be concomitantly studied.

## Data Availability

All data generated or analysed during this study are included in this published article and its supplementary information files.
